# Genome-Wide Identification and Expression Analysis Elucidates the Potential Role of PFK Gene Family in Drought Stress Tolerance and Sugar Metabolism in Cotton

**DOI:** 10.3389/fgene.2022.922024

**Published:** 2022-06-20

**Authors:** Teame Gereziher Mehari, Yanchao Xu, Muhammad Jawad Umer, Fang Hui, Xiaoyan Cai, Zhongli Zhou, Yuqing Hou, Kai Wang, Baohua Wang, Fang Liu

**Affiliations:** ^1^ School of Life Sciences, Nantong University, Nantong, China; ^2^ State Key Laboratory of Cotton Biology, Cotton Institute of the Chinese Academy of Agricultural Sciences, Anyang, China; ^3^ School of Agricultural Sciences, Zhengzhou University, Zhengzhou, China

**Keywords:** cotton, phosphofructokinase, drought stress, sugar metabolism, RNA-Seq, RT-qPCR

## Abstract

Drought has been identified as a major threat for global crop production worldwide. Phosphofructokinase (PFK) is vital for sugar metabolism. During phosphorylation, plants have two enzymes: ATP-dependent phosphofructokinase (PFK) and pyrophosphate-dependent fructose-6-phosphate phosphotransferase (PFP). Genome-wide identification led to the identification of 80 PFK genes, 26 genes in *G. hirsutum* and *G. barbadense*, and 14 genes in *G. arboreum* and *G. raimondii*. Phylogenetic, gene structure, and motif analyses showed that PFK genes were grouped into two main categories, namely, PFK and PFP, with 18 and 8 genes in the allotetraploid species and 10 PFK and 4 PFP genes in the diploid species, respectively. Using the RNA-seq expressions of 26 genes from *GhPFK*, a co-expression network analysis was performed to identify the hub genes. *GhPFK04*, *GhPFK05, GhPFK09*, *GhPFK11*, *GhPFK13*, *GhPFK14*, and *GhPFK17* in leaves and *GhPFK02, GhPFK09*, *GhPFK11*, *GhPFK15*, *GhPFK16,* and *GhPFK17* in root tissues were found as hub genes. RT-qPCR analysis validated the expressions of identified hub genes. Interestingly, *GhPFK11* and *GhPFK17* were identified as common hub genes, and these might be the true candidate genes involved in the drought stress tolerance. In the KEGG enrichment analysis, amino acids such as L-valine, L-histidine, L-glutamine, L-serine, L-homoserine, L-methionine, L-cysteine, and gluconic acid were significantly upregulated, whereas sugars, mainly fructose-1-phosphate, D-mannitol, D-sorbitol, dulcitol, and lactose, were significantly downregulated during drought stress. Genome-wide analysis paves the way for a deeper understanding of the PFK genes and establishes the groundwork for future research into PFK’s role in enhancing drought stress tolerance and sugar metabolism in cotton.

## Introduction

Cotton is an appealing model for investigating polyploid origins, evolution, and domestication. Diversity in *Gossypium* increased by transoceanic, long-distance dispersal and broad hybridization among lineages that are currently geographically separated. *Gossypium hirsutum* L. (AD1) and *Gossypium barbadense* L. (AD2) are two cultivated tetraploids that resulted through transoceanic hybridization (Zhang et al., 2015). They are developed separately and are domesticated in different parts of the world. *G. hirsutum*, which has been domesticated, shows greater adaptability and high production, whereas *G. barbadense* produces uniquely high-quality fibers (Wang et al., 2019). *G. hirsutum* is the main economical essential fiber crop, the primary source of renewable textile fibers, rich in protein and oilseed production. It was one of the first genetically modified crops to be widely used, and human-mediated breeding has resulted in current upland varieties with increased production and fiber quality (Su et al., 2016; Nazir et al., 2020).

Drought stress is a frequent abiotic stress that restricts crop growth and yield around the globe (Yao and Wu, 2016). In plants, drought triggers a complex set of molecular responses that starts with stress detection, progresses through a signal transduction cascade, and ends with physio-morphological changes at the cellular level, such as stomatal closure, cellular respiration activation, and inhibition of cell growth and photosynthesis (Lamaoui et al., 2018; dos Santos et al., 2022). Plants can also generate and accumulate certain metabolites that are specifically engaged in stress tolerance when they are subjected to drought (da Silva et al., 2017).

Glycolysis is a metabolic activity in which enzymes convert glucose into two molecules of pyruvate in the availability of oxygen or two molecules of lactate without oxygen. Anaerobic glycolysis, the latter pathway, is thought to be the first natural method to create adenosine triphosphate (ATP) (Plaxton, 1996). Because mitochondria are absent in few cells, like mature red blood cells, glycolysis is the sole way to produce ATP. It is a cytoplasmic method for converting glucose into two three-carbon molecules, which generates energy. Phosphorylation of glucose with the enzyme hexokinase traps glucose. All cells in the body use it to generate energy. In aerobic situations, glycolysis creates pyruvate, while in anaerobic ones, it produces lactate. To produce extra energy, pyruvate enters the Krebs cycle (Givan, 1999; Ohlendieck, 2010).

The glycolysis and pentose phosphate pathways have a significant impact on crop tolerance and abiotic stress. Phosphofructokinase (PFK) is a range-limiting enzyme in the carbon-flow controlling pathways of biological activities (Yao and Wu, 2016). The phosphorylation of fructose-6-phosphate to fructose-1,6-bisphosphate, a crucial regulatory step, is catalyzed by PFK, a glycolytic enzyme. The transfer of a phosphoryl group from ATP, which is mediated by enzymes, is a vital step in numerous biological activities (Ros and Schulze, 2013).

In crops, phosphofructokinase is vital for sugar metabolism. During phosphorylation, plants have two enzymes such as: ATP-dependent phosphofructokinase (PFK) and pyrophosphate-dependent fructose-6-phosphate phosphotransferase (PFP) (Lü et al., 2019). PFK gene families have been characterized and a number of genes have been identified in some crops, such as eleven genes in *Arabidopsis thaliana* (Mustroph et al., 2007), fifteen PFK genes in rice (Kato-Noguchi, 2002), fourteen genes in white pear (Lü et al., 2019), thirteen genes in cassava (Wang et al., 2021), and studies were also performed in spinach and *Saccharum* (Chen et al., 2017).

The sugar required for root growth and metabolism is transported from leaves, enhancing the sucrose transport from leaves to roots is conducive to maintaining root growth under drought stress (Xu et al., 2015). In line with this, a higher root/shoot (R/S) ratio was pronounced under drought stress in soybeans. It increased the contents of soluble sugar and sucrose in the leaves, but decreased starch content; in the roots, all of these parameters were increased. This may be related to the enhanced carbohydrate metabolism activity under drought stress, including notable changes in the activities of sugar metabolism enzymes and the expression levels of genes in soybeans (Du et al., 2020). Sugar metabolism is an important process for root development under drought stress (Xu et al., 2015; Du et al., 2020).

Drought stress has a negative impact on cotton yield and productivity. Like other abiotic stresses, it is one of the most significant environmental elements influencing cotton production and growth, as well as fiber quality, due to inadequate cellulose cell formation in the bolls (Azhar and Rehman, 2018). Drought has put cotton growers’ future prospects in jeopardy; thus, finding a solution to this challenge is critical (Zahid et al., 2021).

In this research, using published genome as a reference, we performed genome-wide identification and expression analyses on the four cotton species. Phylogenetic tree, gene structure, motif analysis, chromosomal location, gene ontology, subcellular localization, protein–protein interaction, and promoter analysis along with the RNA-seq validation by RT-qPCR analysis were performed to analyze the evolution and potential of the PFK gene family under drought stress tolerance and sugar metabolism. The identified drought stress response genes, as well as the PFK gene family in general, will be used to better understand genomic architecture and functional structure, as well as to characterize the PFK gene family in cotton drought tolerance and sugar metabolism studies.

## Materials and Methods

### Planting Materials and Stress Treatments

The seeds of three cotton species were obtained from the Institute of Cotton Research, Chinese Academy of Agricultural Sciences (Mehari et al., 2021a). The seeds were soaked in water overnight. They were sown for about 7 days in a filter paper until good germination and then transferred to a greenhouse with 16 h of light and 8 h of darkness. When the plants grew to the three true leaves stage, the seedlings were subjected to PEG-6000. The leaf and root tissues at 0 h, 24 h, and 48 h were collected. The samples were promptly frozen in liquid nitrogen and kept at −80°C until the RNA extraction was completed (Mehari et al., 2021b).

### PFK Gene Identification in Cotton

To identify the PFK genes in different cotton genomes, the PF00365 number was used to search the cotton sequence database, and *G. hirsutum* from NAU assembly, *G. barbadense* from HAU assembly, *Gossypium arboreum* from CRI assembly, and *Gossypium raimondii* from JGI assembly were filtered from CottonFGD (http://www.cottonfgd.org/search/) database (Zhu et al., 2017).

### Phylogenetic Analysis of PFK Genes

The ClustalX2 software was used to align the protein sequences of all the identified PFK genes from the four cotton species, that is, *G. hirsutum, G. arboreum, G. barbadense,* and *G. raimondii* as well as *A. thaliana* and *Theobroma cacao* using default parameters. The phylogenetic analysis of PFK from all species was constructed using the numerous sequence alignments imported and displayed in MEGA 7.0 using the neighbor joining approach. For statistical reliability, tree nodes were calculated using the Bootstrap method with 1,000 repeats (Tamura et al., 2011).

### Gene Architecture and Chromosomal Location of PFK Genes

GSDS 2.0 (https://gsds.cbi.pku.edu.cn/) was used to illustrate the gene structure of PFK genes using Genomic DNA and CDS sequences of each species (Hu et al., 2015). The CottonFGD genome annotation files were used to identify the chromosomal locations of PFK genes in *G. hirsutum*, *G. arboreum*, *G. barbadense*, and *G. raimondii*. This information was also used to construct chromosomal mapping which was then displayed by TBtools (Chen et al., 2020). The PFK protein sequences of the four cotton species were submitted to the online motif and domain identification software MEME (http://meme-suite.org/) to determine the conserved domains found in the proteins. The motif search was carried out using a total count of 10 motifs. The MAST tool was used to show the protein database for the identified motifs (Bailey et al., 2009).

### Gene Ontology and Subcellular Localization Prediction

The Gene ontology analysis was performed by using the data downloaded from CottonFGD (www.cottonfgd.org) and the figure was visualized using the graphPad prism program (Zhu et al., 2017). The protein sequences of PFK genes were uploaded to the WoLF PSORT online website (https://wolfpsort.hgc.jp) for predictions of subcellular localization.

### Cis-Regulatory and Protein–Protein Interaction Analysis of PFK Genes

The PlantCARE (http://bioinformatics.psb.ugent.be/webtools/plantcare/html/) online tool was used to search putative cis-regulatory elements in the PFK genes’ promoter regions up to 2,000 bp upstream, which were retrieved from the CottonFGD database (Lescot et al., 2002). The TBtools software was used to create a circular heatmap of cis elements (Chen et al., 2020). The amino acid sequences of the common hub genes *GhPFK11* and *GhPFK17* were used as query sequences to obtain the protein–protein interaction network by using the STRING website (https://cn.string-db.org/).

### Expression Analysis of PFK Genes and RT-qPCR Assay

To study the expression of PFK genes, the gene expression profiles were obtained from previously published RNA-Seq data (NCBI accession: PRJNA663204) in the leaf and root tissues of different *G. hirsutum* races under drought stress. The FPKM values were used to present the PFK expression levels. RNA was extracted by TianGEN kit following the protocol guidelines and was reverse transcribed into cDNA by cDNA synthesis SuperMix for qPCR (one step for gDNA removal). Finally, the SYBR Green SuperMix kit was used to perform quantitative RT-qPCR as directed in the instruction manual. Three technical and biological replications were used in the experiment. The 2^−ΔΔCt^ approach was used to calculate the relative expression data (Schmittgen and Livak, 2008). The primers used in the experiment were designed via NCBI (https://www.ncbi.nlm.nih.gov/) and shown in the Supplementary Table S1.

## Results

### PFK Gene Family Identification in Cotton Species

The CottonFGD database (www.cottonfgd.org) was used to identify the PFK encoding genes in both diploid and tetraploid cotton species. The four cotton species were found to have a total of 80 genes, with 26 genes in *G. hirsutum* and *G. barbadense* and 14 genes in the diploid species of *G. arboreum* and *G. raimondii*. The CDS length of the cotton species ranges between 372 and 1854 bp, 1302 and 1851 bp, 372 and 1851 bp, and 840 and 1854 bp in *G, hirsutum*, *G. barbadense, G. arboreum,* and *G, raimondii,* respectively. Similarly, 299–617 aa, 299–617 aa, 299–617 aa, and 299–617 aa protein length was observed in the above four cotton species successively. Correspondingly, molecular weight distributes between 14.192–67.555 kDa and 47.844–67.38 kDa were recorded in *G. hirsutum* and *G. barbadense*, while 14.208–67.41 kDa and 30.493–67.568 kDa were recorded in both *G. arboreum* and *G. raimondii* successively. In case of molecular charge, similar distributions were observed, −0.5–15, −1–19, −1.5–18, and −3.5–19.5, in *G. hirsutum*, *G. arboreum*, *G. raimondii,* and *G. barbadense,* correspondingly. The GRAVY nature of the four cotton species was low and negative (Tables 1–4). This implies that the PFK proteins are hydrophilic.

**TABLE 1:** Transcript and physiochemical features of PFK genes in *G. hirsutum*.

Transcript ID	Gene name	Chr #	Start	End	Strand	Gene length (bp)	CDS length (bp)	Mean exon length (bp)	Mean intron length (bp)	Protein length (aa)	MW (kDa)	Charge	PI	GRAVY
*Gh_A02G0878.1*	*GhPFPA1*	A02	27983885	27988803	−	4,919	1854	103	180.3	617	67.555	7.5	7.366	−0.146
*Gh_A02G1133.1*	*GhPFK01*	A02	61785439	61788827	−	3,389	1509	116.1	156.7	502	54.868	3.5	6.901	−0.235
*Gh_A02G1250.1*	*GhPFK02*	A02	74165599	74171506	+	5,908	1617	124.4	357.6	538	59.306	11.5	7.849	−0.189
*Gh_A03G1964.1*	*GhPFK03*	Scaffold_A03	150231	150719	+	489	372	186	117	123	14.192	3	8.239	−0.379
*Gh_A05G0198.1*	*GhPFK04*	A05	2069765	2072937	+	3,173	1773	147.8	127.3	590	65.239	9	7.668	−0.263
*Gh_A05G1517.1*	*GhPFK05*	A05	15489354	15490943	−	1,590	1473	736.5	117	490	54.231	7	7.054	−0.231
*Gh_A06G0604.1*	*GhPFK06*	A06	15518034	15521797	+	3,764	1668	128.3	174.7	555	61.379	12.5	8.284	−0.286
*Gh_A06G0820.1*	*GhPFK07*	A06	30143450	30147508	−	4,059	1473	113.3	215.5	490	53.814	2	6.745	−0.288
*Gh_A06G1338.1*	*GhPFPB1*	A06	94604259	94608663	−	4,405	1701	106.3	180.3	566	61.553	−2.5	6.155	−0.111
*Gh_A07G0376.1*	*GhPFK08*	A07	4782896	4788853	+	5,958	1587	113.4	336.2	528	58.204	4.5	7.268	−0.201
*Gh_A07G2294.1*	*GhPFK09*	Scaffold_A07	180176	183360	−	3,185	1449	111.5	144.7	482	53.007	14	8.641	−0.192
*Gh_A11G1421.1*	*GhPFPB3*	A11	19028378	19033169	+	3,904	1710	106.9	205.5	569	62.593	0	6.511	−0.18
*Gh_A13G1730.1*	*GhPFK10*	A13	76200172	76208188	−	4,792	1437	110.5	548.3	478	52.472	9	7.803	−0.189
*Gh_D02G1012.1*	*GhPFPA2*	D02	24830229	24835396	+	8,017	1851	102.8	195.1	616	67.471	8.5	7.524	−0.129
*Gh_D03G0389.1*	*GhPFK11*	D03	5424547	5430634	+	5,168	1617	124.4	372.6	538	59.229	11	7.713	−0.188
*Gh_D03G0556.1*	*GhPFK12*	D03	10902780	10905086	−	6,088	900	128.6	234.5	299	32.918	1	6.666	−0.134
*Gh_D05G0274.1*	*GhPFK13*	D05	2434690	2437805	+	2,307	1725	143.8	126.5	574	63.533	15	8.395	−0.268
*Gh_D05G1688.1*	*GhPFK14*	D05	15175474	15177063	−	3,116	1470	735	120	489	54.166	7	7.116	−0.246
*Gh_D06G0684.1*	*GhPFK15*	D06	11829902	11833654	+	1,590	1668	128.3	173.8	555	61.207	13.5	8.449	−0.305
*Gh_D06G1667.1*	*GhPFPB4*	D06	55410670	55415080	−	3,753	1701	106.3	180.7	566	61.602	0.5	6.582	−0.134
*Gh_D07G0203.1*	*GhPFK16*	D07	2148309	2151465	−	4,411	1449	111.5	142.3	482	53.127	15	8.775	−0.195
*Gh_D07G0439.1*	*GhPFK17*	D07	4733687	4739703	+	3,157	1587	113.4	340.8	528	58.264	4	7.097	−0.178
*Gh_A05G0198.1*	*GhPFK04*	A05	2069765	2072937	+	6,017	1773	147.8	127.3	590	65.239	9	7.668	−0.263
*Gh_D10G0107.1*	*GhPFPB5*	D10	845050	849103	+	4,054	1779	118.6	162.5	592	64.872	−0.5	6.441	−0.086
*Gh_D11G1572.1*	*GhPFPB6*	D11	16161960	16166675	+	4,716	1710	106.9	200.4	569	62.672	4.5	7.044	−0.187
*Gh_D13G2078.1*	*GhPFK18*	D13	56288459	56298463	−	10,005	1431	110.1	714.5	476	52.224	9	7.836	−0.222

Chr #, chromosome number; bp, base pair; aa, amino acid; MW, molecular weight; kDa, kilodalton; pI, iso-electric point; GRAVY, grand average of hydropathy.

**TABLE 2:** Transcript and physiochemical features of PFK genes in *G. arboreum*.

Transcript ID	Gene name	Chr #	Start	End	Strand	Gene length (bp)	CDS length (%)	Mean exon length (bp)	Mean intron length (bp)	Protein length (aa)	MW (kDa)	Charge	PI	GRAVY
*Ga01G2780.1*	*GaPFK01*	A01	112187850	112188338	+	489	372	186	117	123	14.208	3	8.239	−0.335
*Ga02G0430.1*	*GaPFK02*	A02	6567294	6573204	−	5911	1617	124.4	357.8	538	59.306	11.5	7.849	−0.189
*Ga02G0682.1*	*GaPFK03*	A02	26885445	26888798	+	3354	1509	116.1	153.8	502	54.862	5.5	7.182	−0.221
*Ga03G1080.1*	*GaPFPA1*	A03	39283790	39288712	−	4923	1851	102.8	180.7	616	67.41	9.5	7.683	−0.144
*Ga05G0289.1*	*GaPFK04*	A05	2603640	2606732	+	3093	1725	143.8	124.4	574	63.536	12.5	8.227	−0.228
*Ga05G1883.1*	*GaPFK05*	A05	17010181	17011769	−	1589	1470	735	119	489	54.157	4.5	6.852	−0.242
*Ga06G0702.1*	*GaPFK06*	A06	11952219	11955985	+	3767	1668	128.3	174.9	555	61.365	12.5	8.284	−0.286
*Ga06G1021.1*	*GaPFK07*	A06	33241527	33245603	−	4077	1473	113.3	217	490	53.828	2	6.745	−0.288
*Ga06G1856.1*	*GaPFPB1*	A06	119558833	119563236	−	4404	1701	106.3	180.2	566	61.49	−0.5	6.441	−0.114
*Ga07G0263.1*	*GaPFK08*	A07	2791986	2795312	−	3327	1581	121.6	145.5	526	58.044	19	9.126	−0.194
*Ga07G0524.1*	*GaPFK09*	A07	5587128	5593089	+	5962	1587	113.4	336.5	528	58.111	3.5	7.065	−0.193
*Ga10G2984.1*	*GaPFPB2*	A10	128611006	128615063	−	4058	1701	106.3	157.1	566	61.874	−1	6.383	−0.16
*Ga11G2398.1*	*GaPFPB3*	A11	104247268	104252050	−	4783	1710	106.9	204.9	569	62.939	2.5	6.787	−0.191
*Ga13G2421.1*	*GaPFK10*	A13	119436074	119444081	−	8008	1437	110.5	547.6	478	52.444	9	7.803	−0.19

Chr #, chromosome number; bp, base pair; aa, amino acid; MW, molecular weight; kDa, kilodalton; pI, iso-electric point; GRAVY, grand average of hydropathy.

**TABLE 3:** Transcript and physiochemical features of PFK genes in *G. raimondii*.

Transcript ID	Gene name	Chr #	Start	End	Strand	Gene length (bp)	CDS length (%)	Mean exon length (bp)	Mean intron length (bp)	Protein length (aa)	MW (kDa)	Charge	PI	GRAVY
*Gorai.001G025000.1*	*GrPFK01*	D01	2353903	2357870	−	3968	1647	175.2	140.8	548	60.768	18	8.568	−0.195
*Gorai.001G051000.1*	*GrPFK02*	D01	4832464	4839210	+	6747	1587	167	339.2	528	58.207	5	7.286	−0.163
*Gorai.003G042800.1*	*GrPFK03*	D03	5450969	5457737	+	6769	1617	178.6	370.6	538	59.229	11	7.713	−0.188
*Gorai.003G061100.1*	*GrPFK04*	D03	10796852	10799714	−	2863	1257	104.8	146	418	46.243	6.5	7.363	−0.155
*Gorai.005G115600.1*	*GrPFPA1*	D05	22599435	22605464	+	6030	1854	146.2	195.1	617	67.568	8.5	7.524	−0.142
*Gorai.007G170900.1*	*GrPFPB1*	D07	15439718	15444527	+	4810	1707	108.7	204.7	568	62.573	4.5	7.044	−0.195
*Gorai.009G029100.1*	*GrPFK05*	D09	2226642	2230570	+	3929	1725	177.8	134.8	574	63.604	15	8.395	−0.256
*Gorai.009G185000.1*	*GrPFK06*	D09	14234340	14236341	−	2002	1470	941	120	489	54.191	6.5	7.032	−0.244
*Gorai.010G079100.1*	*GrPFK07*	D10	11493546	11498094	+	4549	1668	190	173.3	555	61.203	13.5	8.507	−0.302
*Gorai.010G103800.1*	*GrPFK08*	D10	18880342	18883026	+	2685	840	93.3	230.6	280	30.493	11.5	9.11	−0.331
*Gorai.010G103900.1*	*GrPFK09*	D10	18886681	18891336	−	4656	1473	159.4	215.3	490	53.641	1	6.624	−0.272
*Gorai.010G184300.1*	*GrPFPB2*	D10	53477489	53482966	−	5478	1701	179.5	173.7	566	61.567	−1.5	6.302	−0.136
*Gorai.011G011600.1*	*GrPFPB3*	D11	820017	824604	+	4588	1701	145.7	150.5	566	61.851	−1.5	6.303	−0.165
*Gorai.013G229200.1*	*GrPFK10*	D13	54804614	54812629	−	8016	1431	113.2	545.3	476	52.266	9	7.836	−0.201

Chr #, chromosome number; bp, base pair; aa, amino acid; MW, molecular weight; kDa, kilodalton; pI, iso-electric point; GRAVY, grand average of hydropathy.

**TABLE 4:** Transcript and physiochemical features of PFK genes in *G. barbadense*.

Transcript ID	Gene name	Chr #	Start	End	Strand	Gene length (bp)	CDS length (%)	Mean exon length (bp)	Mean intron length (bp)	Protein length (aa)	MW (kDa)	Charge	PI	GRAVY
*Gbar_A02G009620.1*	*GbPFPA1*	A02	33078079	33083928	−	5850	1851	154.6	180.5	616	67.38	9.5	7.683	−0.137
*Gbar_A02G012760.1*	*GbPFK01*	A02	76466974	76470364	−	3391	1590	122.3	150.1	529	57.77	7	7.327	−0.182
*Gbar_A02G014220.1*	*GbPFK02*	A02	90947968	90954531	+	6564	1617	174.8	357.7	538	59.292	11.5	7.849	−0.19
*Gbar_A05G002520.1*	*GbPFK03*	A05	2313058	2316747	+	3690	1725	157.9	136.4	574	63.523	12.5	8.227	−0.237
*Gbar_A05G017640.1*	*GbPFK04*	A05	16387664	16389252	−	1589	1422	474	83.5	473	52.463	7	7.116	−0.245
*Gbar_A06G007200.1*	*GbPFK05*	A06	15846931	15851523	+	4593	1668	192.2	174.6	555	61.379	12.5	8.284	−0.286
*Gbar_A06G009460.1*	*GbPFK06*	A06	30004949	30009643	−	4695	1473	162.1	215.7	490	53.828	2	6.745	−0.288
*Gbar_A06G016360.1*	*GbPFPB1*	A06	105197849	105203545	−	5697	1701	189.1	178.1	566	61.553	−2.5	6.155	−0.111
*Gbar_A07G002540.1*	*GbPFK07*	A07	2781640	2785230	−	3591	1647	142.6	144.8	548	60.514	19.5	9.033	−0.166
*Gbar_A07G004730.1*	*GbPFK08*	A07	5531897	5538486	+	6590	1587	158.5	336.2	528	58.204	4.5	7.268	−0.201
*Gbar_A10G001200.1*	*GbPFPB2*	A10	936595	941048	+	4454	1302	235.7	115.8	433	47.844	−3.5	5.878	−0.196
*Gbar_A11G015950.1*	*GbPFPB3*	A11	18500653	18505440	+	4788	1707	106.7	205.4	568	62.624	0	6.511	−0.183
*Gbar_A13G021230.1*	*GbPFK09*	A13	104899524	104907657	−	8134	1431	119.1	548.8	476	52.286	9	7.836	−0.208
*Gbar_D02G010860.1*	*GbPFPA2*	D02	23684632	23690730	+	6099	1740	156.4	193.2	579	63.736	8.5	7.524	−0.171
*Gbar_D03G004100.1*	*GbPFK10*	D03	5429725	5436416	+	6692	1632	172.4	370.9	543	59.869	12.5	7.836	−0.174
*Gbar_D05G002800.1*	*GbPFK11*	D05	2438237	2441342	+	3106	1449	129.3	118.8	482	52.956	2.5	6.828	−0.226
*Gbar_D05G018110.1*	*GbPFK12*	D05	15588556	15590428	−	1873	1470	876.5	120	489	54.218	7.5	7.138	−0.239
*Gbar_D06G007450.1*	*GbPFK13*	D06	11808443	11812989	+	4547	1617	185.3	178.2	538	59.292	11	8.203	−0.284
*Gbar_D06G009890.1*	*GbPFK14*	D06	19029717	19033756	+	4040	1473	113.3	213.9	490	53.631	1	6.624	−0.271
*Gbar_D06G009900.1*	*GbPFK15*	D06	19037339	19041938	−	4600	1473	156.4	213.9	490	53.631	1	6.624	−0.271
*Gbar_D06G017040.1*	*GbPFPB4*	D06	54055440	54060921	−	5482	1698	173.4	180.5	565	61.465	0	6.512	−0.128
*Gbar_D07G002510.1*	*GbPFK16*	D07	2536630	2540438	−	3809	1581	161.8	142.2	526	58.047	19	9.14	−0.197
*Gbar_D07G005030.1*	*GbPFK17*	D07	5209039	5215828	+	6790	1692	176.1	332.7	563	62.433	5.5	7.302	−0.104
*Gbar_D10G001120.1*	*GbPFPB5*	D10	824918	829627	+	4710	1701	147.4	156.8	566	61.853	−1.5	6.303	−0.156
*Gbar_D11G016720.1*	*GbPFPB6*	D11	16179099	16183855	+	4757	1707	108.9	201	568	62.601	4.5	7.044	−0.191
*Gbar_D13G021590.1*	*GbPFK18*	D13	55974312	55982847	−	8536	1479	144.4	501.2	492	54.037	8	7.624	−0.234

Chr #, chromosome number; bp, base pair; aa, amino acid; MW, molecular weight; kDa, kilodalton; pI, iso-electric point; GRAVY, grand average of hydropathy.

### Phylogenetic Analysis of PFK Proteins

**FIGURE 1:**
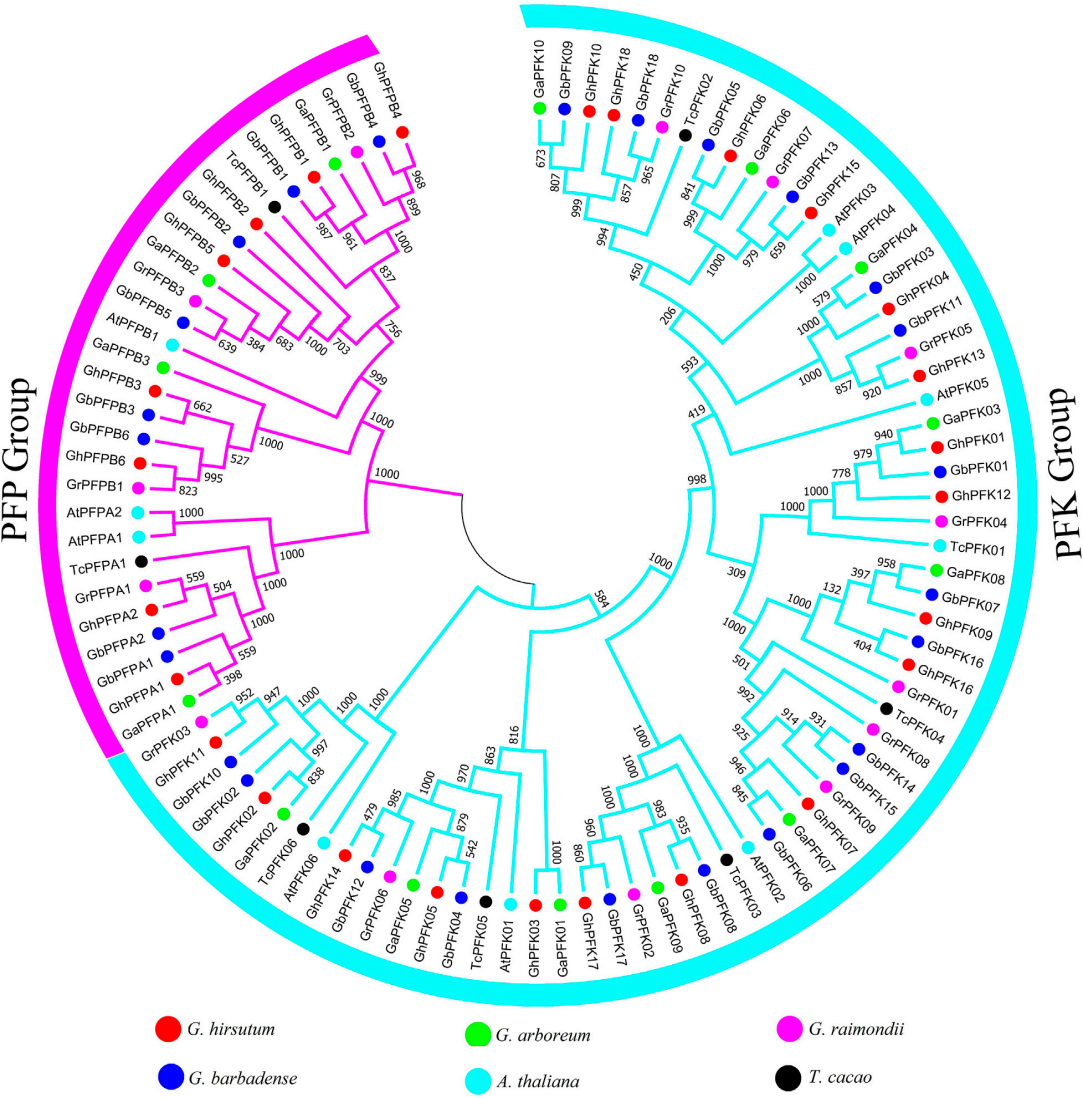
Phylogenetic tree analysis of PFK genes in *G. hirsutum*, *G. barbadense*, *G. arboreum*, *G. raimondii*, *A. thaliana,* and *T. cacao*. The tree analysis categorized the PFK gene family into two big groups of PFK and PFP which are highlighted in blue and violet colors.

The evolution analysis and patterns of cotton PFK proteins were exhibited by building a phylogenetic tree in *G. hirsutum*, *G. barbadense*, *G. arboreum*, *G. raimondii*, *A. thaliana,* and *T. cacao*. The phylogenetic tree of PFK proteins was composed of two main categories, namely, PFK and PFP group (Figure 1). To construct the tree, ClustalX was used to align 97 protein sequences from *G. hirsutum*, *G. barbadense*, *G. arboreum*, *G. raimondii*, *A. thaliana*, and *T. cacao*. From this, 18 PFK and 8 PFP proteins each from *G. hirsutum* and *G. barbadense*, 10 PFK, and 4 PFP proteins from *G. arboreum* and *G. raimondii*, 7 PFK with 2 PFP from *A. thalian*a and 6 PFK with 2 PFP from *T. cacao* were used to build the tree via MEGA 7.0.

### Genetic Architecture and Motif Analysis

**FIGURE 2:**
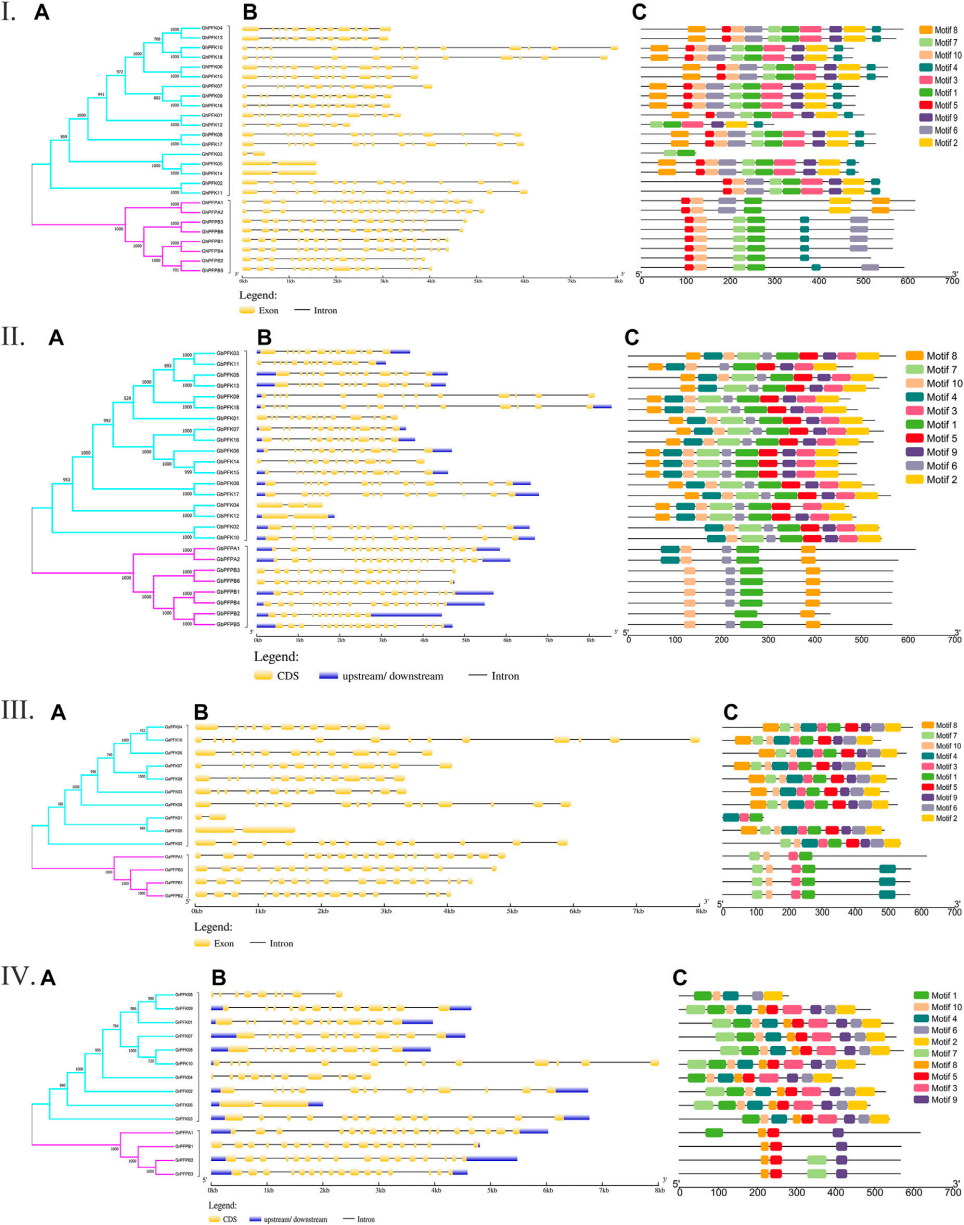
Gene architecture and motif enrichment analysis of PFK proteins in *Gossypium* species. I. *G. hirsutum;* II. *G. barbadense;* III. *G. arboreum;* IV. *G. raimondii*. **(A–C)** stands for phylogenetic relationship, gene structure, and motif identification in all the *Gossypium* species, respectively.

The genetic architecture of PFK proteins was demonstrated by studying the exon-intron structural distribution in the four cotton species. The lowest exon number was recorded in genes *GaPFK01* and *GaPFK05* in *G. arboreum*, *GrPFK06* in *G. raimondii*, *GhPFK03*, *GhPFK05*, and *GhPFK14* in *G. hirsutum*, and *GbPFK12* in *G. barbadense* with 2 exons while the highest number of exons “18” was present in *GaPFPA1* in *G. arboreum*, *GrPFPA1* in *G. raimondii*, *GhPFPA1* and *GhPFPA2* in *G. hirsutum*, *GbPFPA1* and *GbPFPA2* in *G. barbadense*. In relative to gene length, 10,005 bp, 8536 bp, 8008 bp, and 8016 bp were observed in *GhPFK18*, *GbPFK18*, *GaPFK10,* and *GrPFK10* in the four species, respectively (Figure 2A). This showed us the lowest exon numbers in PFK gene group and the highest exon numbers which harbors the PFP gene group. By analyzing the protein sequences of the PFK gene family using the MEME online tool, 10 conserved motifs were predicted. The identified motifs varied between 6 and 10 in *G. hirsutum*, 3 and 10 in *G. arboreum*, 3 and 10 in *G. raimondii,* and 5 and 10 in *G. barbadense*. The lowest number of motifs were observed in *GaPFK01* with motif numbers of 4, 3, and 1 and *GrPFPB1* with 8, 5, and 9 motifs. The PFP subgroup has lower number of motifs between 3 and 6, with motif number 1 as a common motif (Figure 2B). Generally, the FPK proteins have highly conserved motifs and as a result, proteins with comparable structures are likely to have similar functional tasks.

### Chromosomal Mapping of PFK Genes

**FIGURE 3:**
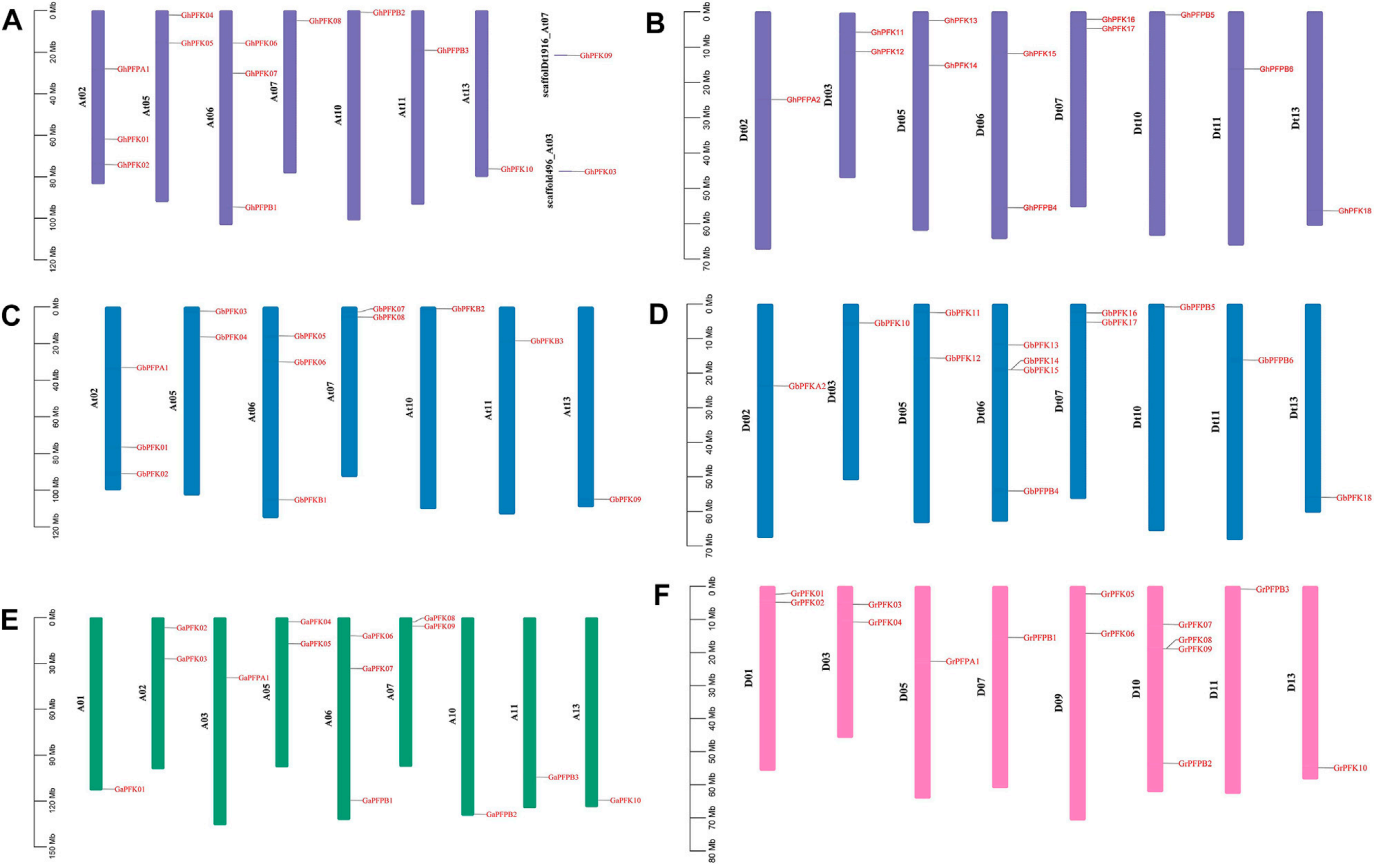
Chromosomal location of the PFK genes. **(A)** *G. hirsutum* At subgenome; **(B)** *G. hirsutum* Dt subgenome; **(C)** *G. barbadense* At subgenome; **(D)** *G. barbadense* Dt subgenome; **(E)** *G. arboreum;* **(F)** *G. raimondii*.

The distribution of PFK genes on chromosomes varies between the tetraploid and diploid cotton species. In the allotetraploid species of *G. hirsutum* and *G. barbadense*, At01, At03, At04, At08, At09, At12 from At subgenome and Dt01, Dt04, Dt08, Dt09, and Dt12 from Dt subgenome had no genes on any chromosome. The gene distribution was between 1 and 4 genes among the rest 15 chromosomes with two scaffolds on the *G. hirsutum* At subgenome (Figure 3). Similarly, in the diploid cotton species of *G. arboreum* and *G. raimondii*, A04, A08, A09, A12 and D02, D04, D06, D08, D12 chromosomes did not possess any PFK genes. The PFK genes were irregularly distributed among the 9 and 8 chromosomes of *G. arboreum* and *G. raimondii*, successively between 1 and 4 genes (Figures 3A–F).

### Gene Ontology Enrichment Analysis

**FIGURE 4:**
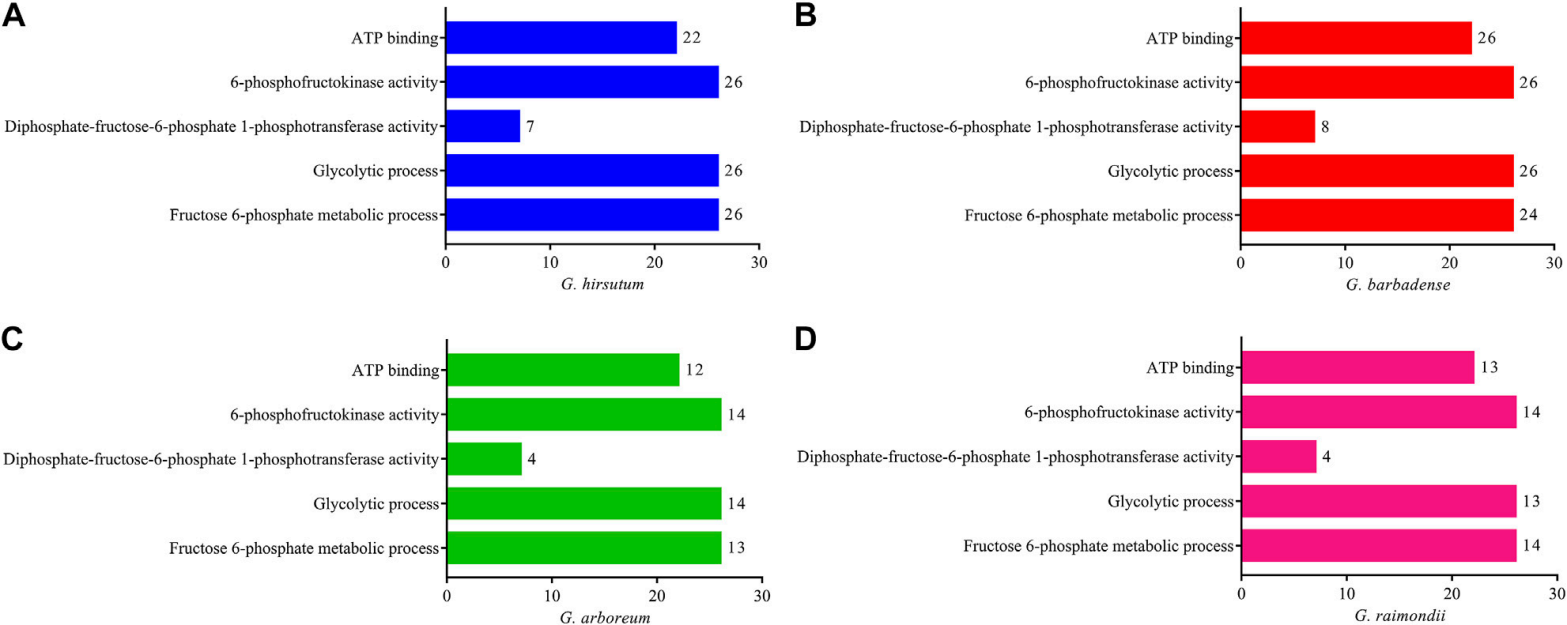
Gene ontology enrichment analysis using CottonFGD for the PFK gene family. **(A)** *G. hirsutum;* **(B)** G. *barbadense;* **(C)** *G. arboreum;* **(D)** *G. raimondii.* Bar graph was done using PrismPad; the scale indicates the number of genes enriched in each function.

Gene ontology enrichment studies were performed on the genes of *G. hirsutum*, *G. barbadense*, *G. arboreum*, and *G. raimondii* to better realize the functional annotations of PFK family genes in *Gossypium* species. Gene ontology analysis proved their glycolytic process (79 genes) and fructose-6-phosphate metabolic process (77 genes) as biological function and 6-phosphofructokinase activity (80 genes), diphosphate-fructose-6-phosphate 1-phosphotransferase activity (23 genes), and ATP binding (73 genes) in the molecular function category in all PFK genes of the *Gossypium* species (Figure 4).

### Subcellular Localization Prediction of PFK Proteins

**FIGURE 5:**
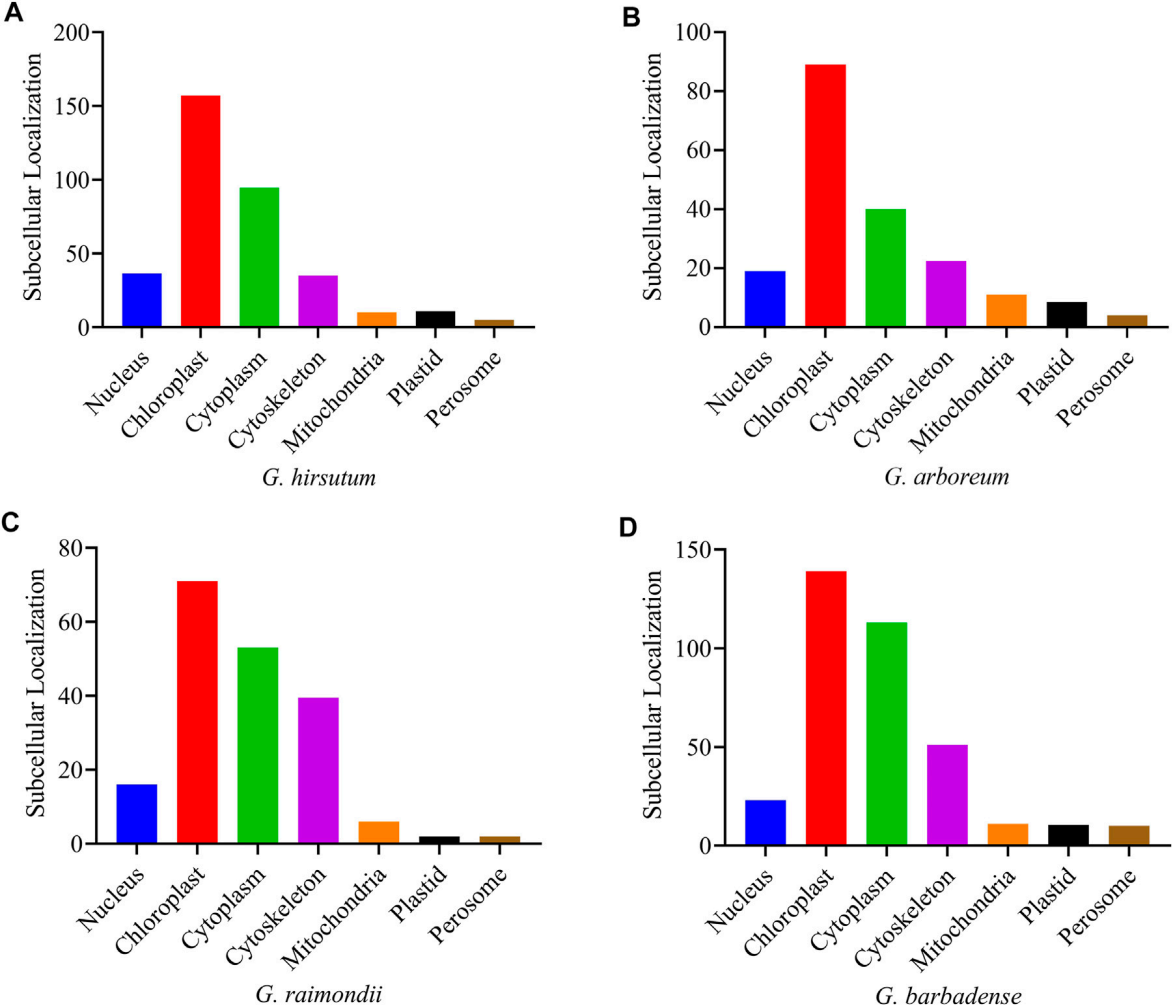
Subcellular localization prediction of the PFK genes of *Gossypium* species **(A)** *G. hirsutum;* **(B)** *G. arboreum;* **(C)** *G. raimondii;* **(D)** *G. barbadense.*

According to the online subcellular localization prediction in the WoLF PSORT (https://wolfpsort.hgc.jp/) tool, the four *Gossypium* species were expected to be localized in various cell sections (Supplementary Table S2), primarily in the chloroplast, cytoplasm, cytoskeleton, and nucleus. A small number of genes were also found in the mitochondria, plastids, and perisome cells (Figure 5).

### Cis-Regulatory Element Analysis

**FIGURE 6:**
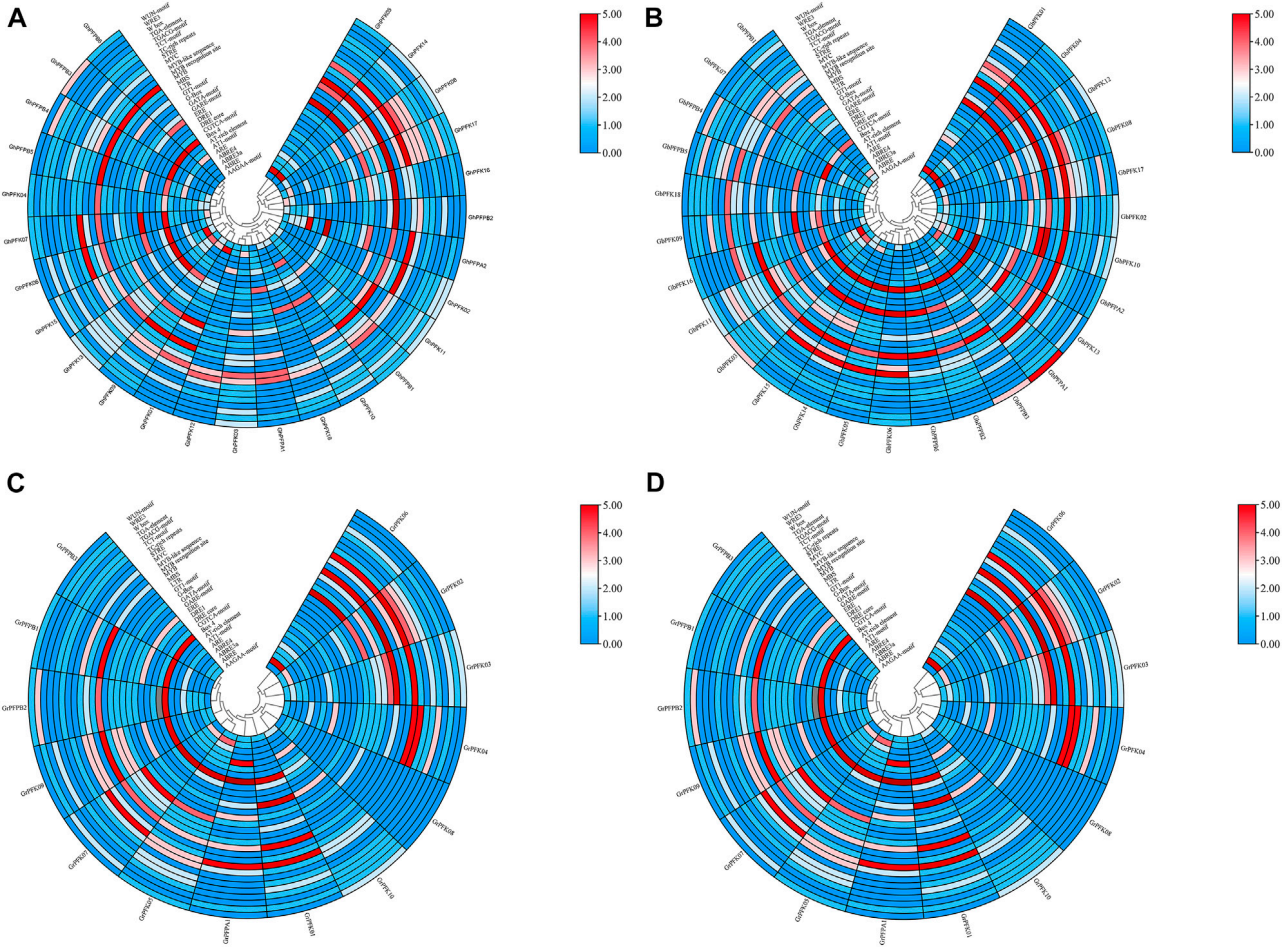
Cis-regulatory elements analysis of the PFK gene family. **(A)** *G. hirsutum;* **(B)** *G. barbadense;* **(C)** *G. arboreum;* **(D)** *G. raimondii*.

CottonFGD (www.cottonfgd.org) was used to identify cis-regulatory components in the 2,000 base pair of the 5′ upstream region. The findings revealed that cis-acting components fall into numerous categories such as defense and stress responsiveness, abscisic acid responsiveness, light responsiveness, anaerobic induction elements, MeJA-responsiveness, hormone-responsive elements, low-temperature responsiveness, and in drought inducibility elements were identified in the promoter regions of both the tetraploid and diploid cotton species (Figure 6, Supplementary Table S3). In addition, the promoter regions of *G. hirsutum*, *G. barbadense*, *G. arboreum*, and *G. raimondii* contained five elements important in drought and stress reactivity, including TC-rich repeats, MBS, ABRE, ARE, and WUN-motif.

### Protein–Protein Interaction Analysis of PFK Genes

**FIGURE 7:**
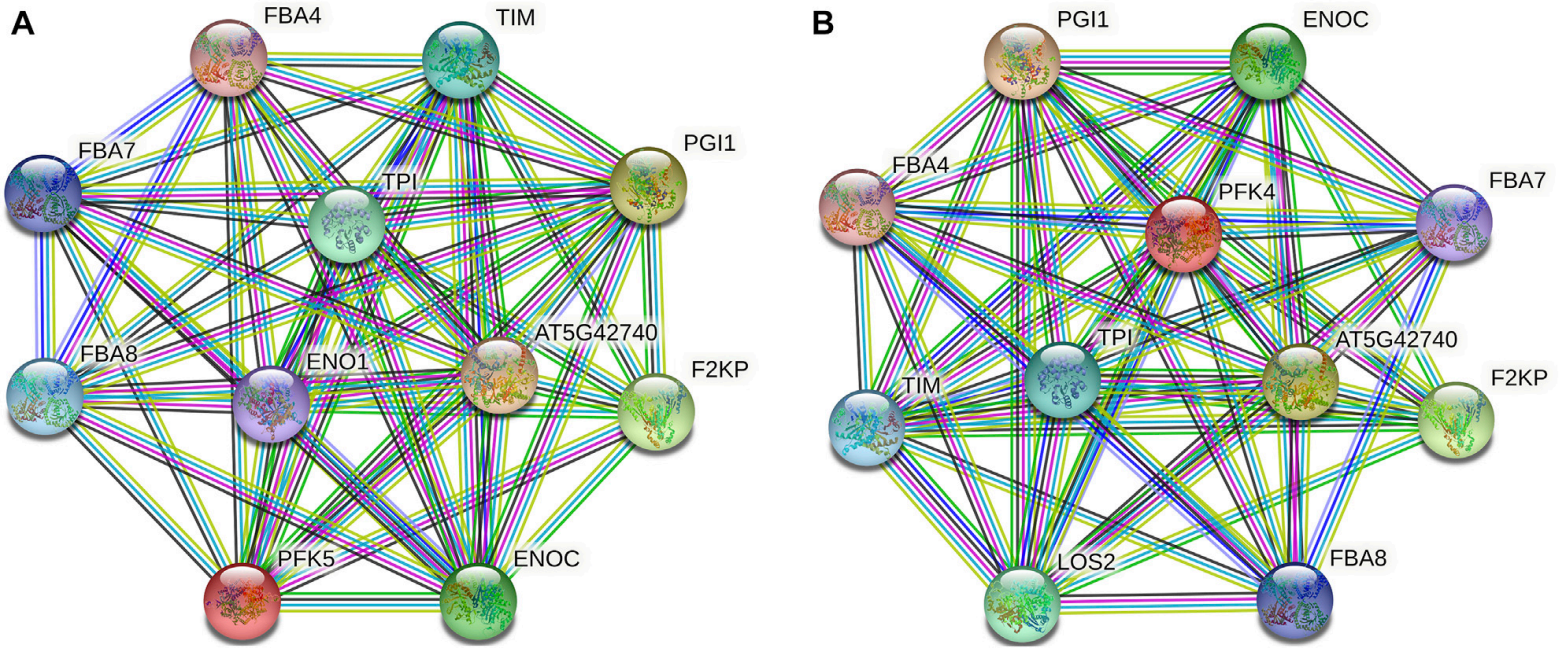
Protein–protein interaction analysis of PFK hub proteins. **(A)**. Interaction network of *GhPFK11* gene; **(B)**. Interaction network of *GhPFK17* gene; Colored nodes: query proteins and first shell of interactors; Red lines: gene fusions; Green lines: gene neighborhood; Black lines: co-expression; Blue lines: gene co-occurrence.

Protein–protein interaction analysis was used to further identify the potential biological activities of PFK hub genes, and 10 putative interactors were discovered (Figure 7). Notably, several sugar metabolism-related proteins that play a key role in glycolysis and gluconeogenesis (FBA4, FBA7, and FBA8) were found. *AT5G42740* and PGI1 encode glucose-6-phosphate and plastid phospho-glucose isomerase which encode for defense response and accumulation of starch in root cap cells. TIM and TPI proteins also encode plastidic triose phosphate and triosephosphate isomerase enzymes.

### RT-qPCR and Gene Co-Expression Network Analysis

**FIGURE 8:**
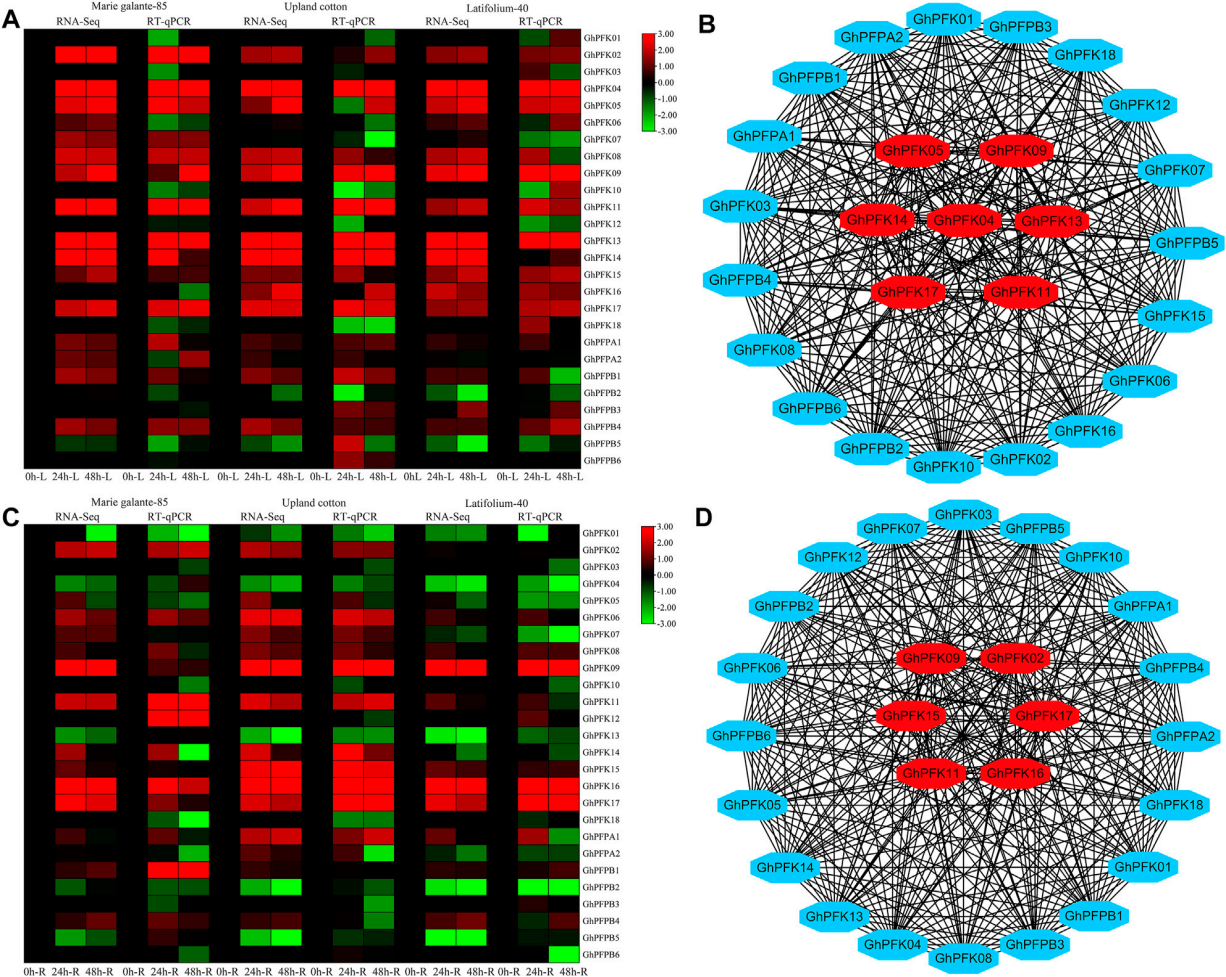
RNA-seq, RT-qPCR, and Co-expression network analysis of PFK genes. **(A)**. RNA-seq and RT-qPCR expression analysis of the PFK genes in the leaf tissue of *G. hirsutum* races; **(B)**. Co-expression analysis and hub gene identification from leaf expression; **(C)**. RNA-seq and RT-qPCR expression analysis of the PFK genes in the root tissue of *G. hirsutum* races; **(D)**. Co-expression analysis and hub gene identification from leaf expression. Genes in the center of the network analysis with red color are hub genes.

Transcriptome analysis was performed in *G. hirsutum* races to observe the expression potential of PFK genes in response to drought stress tolerance. The results of the RNA-seq revealed that in both leaf and root tissues the genes of PFK family showed higher expressions in response to drought stress. *GhPFK02*, *GhPFK04*, *GhPFK05*, *GhPFK09*, *GhPFK11*, *GhPFK13*, *GhPFK14*, and *GhPFK17* in the leaf tissue, *GhPFK02*, *GhPFK06*, *GhPFK09*, *GhPFK11*, *GhPFK15*, *GhPFK16,* and *GhPFK17* in the root tissue showed consistent upregulation in all the three cotton races at all time points (Figures 8A,C). To validate the transcriptome results, RT-qPCR analysis was performed in 26 PFK genes in similar tissues and time points as the transcriptome. The RT-qPCR analysis verified a similar trend of expression with high correlation (R^2^ = 77.5% in the leaf and R^2^ = 64.9% in the root) of both transcriptomes and RT-qPCR analysis results.

For gene co-expression network analysis, a total of 26 genes were used from the PFK gene family. We used the RNA-sequencing data from both leaf and root tissues of *G. hirsutum* races. A correlation analysis of these genes was performed via a correlation calculator. After evaluating the correlation of the genes, the co-expression network analysis was established by cytoscape v.3.7.2 (Shannon et al., 2003). In both leaf and root tissues, 26 nodes (genes) and 325 edges (correlation) were observed. In leaf tissue expressions, there were 191 positive and 134 negative correlations and in root tissue expressions 174 positive and 151 negative correlations were identified. Results indicated that *GhPFK04*, *GhPFK05, GhPFK09*, *GhPFK11*, *GhPFK13*, *GhPFK14*, and *GhPFK17* were found as hub genes in leaves. Similarly, in roots, *GhPFK02, GhPFK09*, *GhPFK11*, *GhPFK15*, *GhPFK16,* and *GhPFK17* were found as hub genes via the cytohubba analysis (Figures 8B,D. Supplementary Table S4). Surprisingly we found that *GhPFK11* and *GhPFK17* are common hub genes in both leaves and roots.

### Metabolite Enrichment Analysis

**FIGURE 9:**
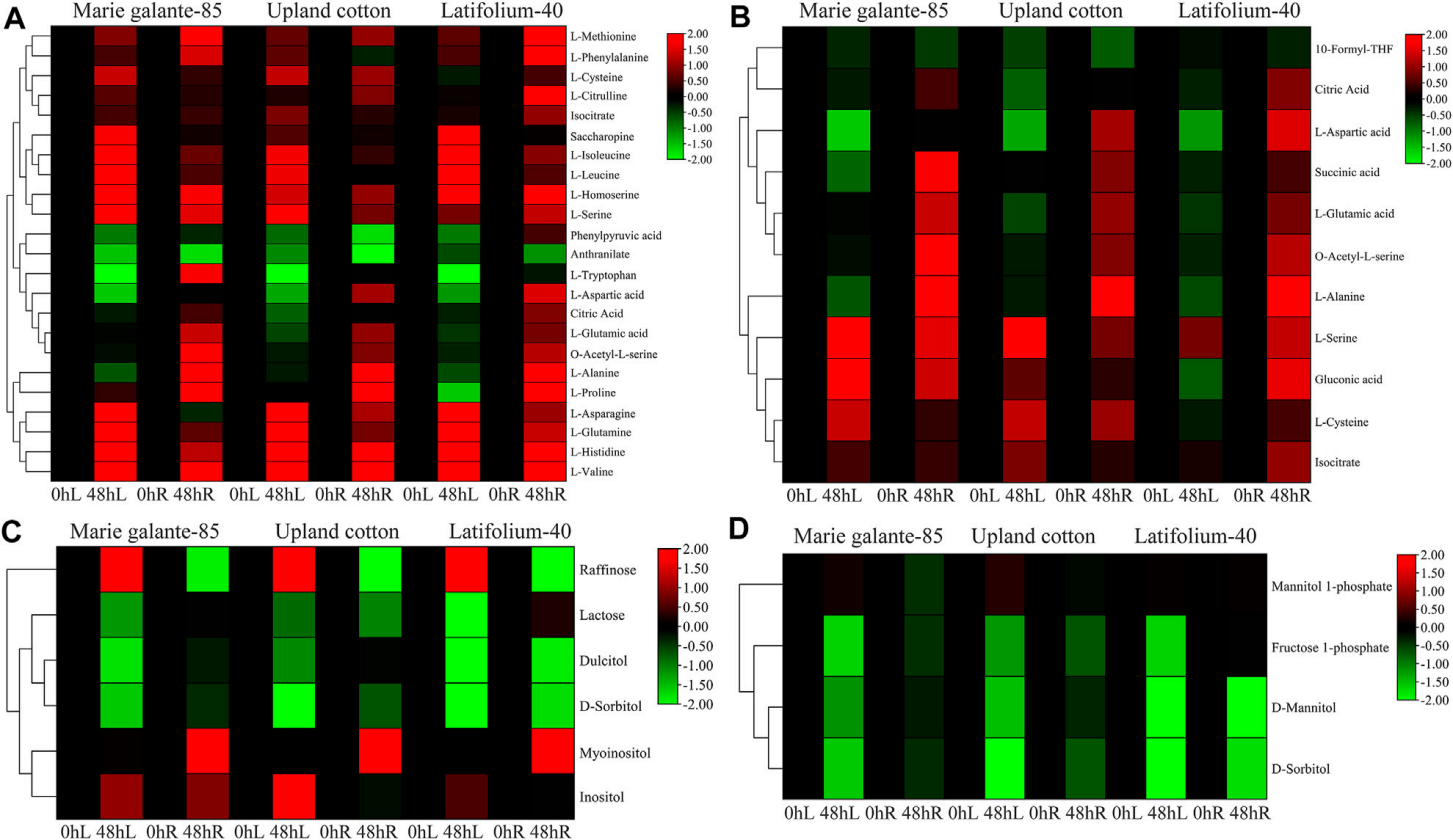
Metabolite enrichment analyses of PFK genes during drought stress. **(A)**. Biosynthesis of amino acids; **(B)**. Carbon metabolism; **(C)**. Galactose metabolism; **(D)**. Fructose and mannose metabolism. Heatmap was visualized by TBtools using the log2 data normalization of the metabolite expression.

KEGG enrichment analysis was performed in the *G. hirsutum* races to identify the significant metabolites that are crucial for drought stress tolerance. Biosynthesis of amino acids, carbon metabolism, fructose and mannose metabolism, and galactose metabolism were all shown to be significantly enriched for the PFK genes (Supplementary Table S5). In the biosynthesis of amino acids and carbon metabolism, most of the amino acids like L-valine, L-histidine, L-glutamine, L-serine, L-homoserine, L-methionine, L-cysteine, and gluconic acid were significantly upregulated, whereas in fructose and mannose, metabolism and galactose metabolism sugars mainly fructose-1-phosphate, D-mannitol, D-sorbitol, dulcitol, and lactose were significantly downregulated under drought stress (Figure 9). In summary, amino acids were upregulated, whereas sugars were downregulated under drought stress treatment.

## Discussion

Cotton (genus *Gossypium*) is a valuable crop that produces the majority of the world’s natural fiber. It is a significant crop that has seen substantial production increases during the last century (Aslam et al., 2020; Grover et al., 2020). *Gossypium* contains more than 50 wild species that are related to cultivated cottons. These species are divided into groups of nearly related species arranged “A-G″ and “K” for diploids and “AD” for tetraploids, and they could provide biotic and abiotic stress tolerance. (Wendel and Grover, 2015; Li et al., 2021). In this study, we used *G. arboreum* and *G. raimondii* from the diploid species category of A and D genomes and *G. barbadense* and *G. hirsutum*, belonging to the AD-genome tetraploid category (Zhang et al., 2008). *G. barbadense* and *G. hirsutum* now account for >95% of the world’s cotton production (Fang et al., 2014; Li et al., 2015).

In the four *Gossypium* species, we identified a total of 80 PFK genes with a majority of PFK (56 genes) and 24 genes from PFP subfamily. The PFP gene subfamily was also categorized as PFPA (Alpha) with 6 genes and PFPB (Beta) with 18 genes. Similarly, in other researched crops, the gene family was divided between PFK and PFP groups. The PFK gene family has eleven members in *A. thaliana*: four belongs to *AtPFP* and seven to *AtPFK.* Rice has fifteen PFK genes, five of which belong to *OsPFP* and ten to *OsPFK*. The white pear (*Pyrus bretschneideri*) has fourteen members in the PFK family, ten of which are *PbPFK* and four of which are *PbPFP*. PFK is a crucial protein in plant growth and development with a wide range of functions, with 13 members of the PFK family in cassava (*Manihot esculenta* Crantz) (Wang et al., 2021).

According to a subcellular localization analysis of PFK genes, five *MePFKs* in chloroplast, two in cytoplasm, and four *MePFPs* were predicted to be localized at the cytoplasm in cassava (*M. esculenta* Crantz). *MePFK03* and *MePFPA1*, which are expected to be found in the chloroplast and cytoplasm, successively, were chosen to generate GFP fusion proteins to support the abovementioned conclusion. GFP images revealed that they were present in the chloroplast and cytoplasm, successively (Wang et al., 2021).

The discovery of cis-regulatory elements unveiled that the PFK genes contain several essential cis-regulatory elements that protect cells from harmful environmental stimuli. Box 4, CGTCA-motif, DRE1, DRE core, ERE, GARE-motif, G-Box, GATA-motif, GT1-motif, LTR, MBS, MYB-like sequence, MYB recognition site, MYB, MYC, STRE, W-box, WRE3, and WUN-motif were some of the vital regulatory elements identified in the PFK family. OMT genes contain W-box, MYB, MYC, DRE, ABRE, G-Box, and MBS. The W-box regulates gene expression and binds WRKY transcription factors. Plants need WRKY TFs to protect themselves from drought, chilling, wounding, heat, and salinity stress (Hafeez et al., 2021).

Cis-regulatory elements belong to numerous classifications such as stress responsive, hormone responsive, and light responsive. They were encoded in the promoter regions of *G. hirsutum*, *G. arboreum*, *G. raimondii*, and *G. barbadense*. Furthermore, four abiotic stress-responsive promoter elements were found in two diploid and two tetraploid cotton species, including LTRs, TC-rich repeats, MBSs, and WUN motifs confirming their role in abiotic stress tolerance (Rehman et al., 2021). ABRE, CGTCA-motif, TCA-element, TGACG-motif, GARE-motif, TATC-box, P-box, and other phytohormone linked abscisic acid, gibberellin, auxin, methyl jasmonate (MeJA), and salicylic acid were identified in the SOD family of *Brassica napus* in a similar study. Stress response (drought, cold, light, and anaerobic) elements such as ARE, chs-CMA1a, LAMP-element, LTR, MBS, TCT-motif, G-box, GT1-motif, and MBS were also found (Su et al., 2021).

Drought is one such negative environmental cue that hinders photosynthetic carbon fixation and impacts sugar transport by reducing the cellular osmotic potential. Sugar transporters are a group of proteins that aid in the transit of sugar within cells. The influx/outflow of different sugars and their metabolite intermediates that enable plant growth and development is determined by these transporter proteins. Sugar distribution across the cellular and subcellular compartments is reprogrammed as a result of abiotic stress, particularly drought stress-induced damage (Kaur et al., 2021).

Glycolysis is a metabolic process that converts glucose into pyruvate in the availability of oxygen or lactate molecules without oxygen via enzyme-catalyzed processes. Anaerobic glycolysis, the latter pathway, is thought to be the first natural method to create adenosine triphosphate (ATP) (Mayes and Bender, 2003). It is a crucial metabolic mechanism in all living things. Glycolysis helps cells adjust to abiotic conditions including lack of oxygen, cold, and drought by providing energy for cell and sugar metabolism (Wang et al., 2021).

Sugars are plants’ basic products, and the hexose sugars glucose and fructose provide the starting material for almost all metabolic pathways in crops. Hexose sugars are necessary to be phosphorylated before they can be digested. Hexokinases and fructokinases are the only two families of enzymes found in plants that catalyze the necessary permanent phosphorylation of glucose and fructose. These enzymes appear to coordinate sugar synthesis with water, sunlight, nutrients, and CO_2_ absorption (Granot et al., 2014). In plants, fructose was formed as a breakdown product of both primary sucrose-cleaving enzymes. A fructokinase phosphorylates fructose before it enters metabolism. The phosphofructokinase B family, which includes known FRKs, is a heterogeneous group of carbohydrate/purine kinases (Riggs et al., 2017). The primary step of fructose phosphorylation in plants is catalyzed by two types of fructokinases: cytosolic and plastidic fructokinases (Stein et al., 2016). Fructokinases have an impact on long-lasting developmental processes such as sugar, nutrients, and water transport (Granot et al., 2014).

Metabolite enrichment analysis was used to directly investigate a group of functionally related metabolites that are significantly enriched. Biosynthesis of amino acids, carbon metabolism, fructose and mannose metabolism, and galactose metabolism were significantly enriched during drought stress. Many amino acids response to abiotic stressors have been discovered to play vital functions in plant growth. Amino acids also serve as precursors for a variety of primary and secondary metabolites, and they play an important role in human nutrition (Trovato et al., 2021). In both normally watered and drought-stressed plants, exogenous addition of amino acids can improve nutritional traits like phenol, total protein, proline, and several important amino acids like glutamic acid, glutamine, and asparagine. By reducing the negative effects of drought stress on cabbage, amino acids were found to be efficient in boosting physiological and nutraceutical parameters. Thus, the use of amino acids proved successful in reducing the impacts of drought stress and boosting nutritional value in drought stress situations (Haghighi et al., 2020). Overall, The above results widen our understanding about the role of *Gossypium* PFK genes on drought stress tolerance and sugar metabolism.

## Conclusions

**FIGURE 10:**
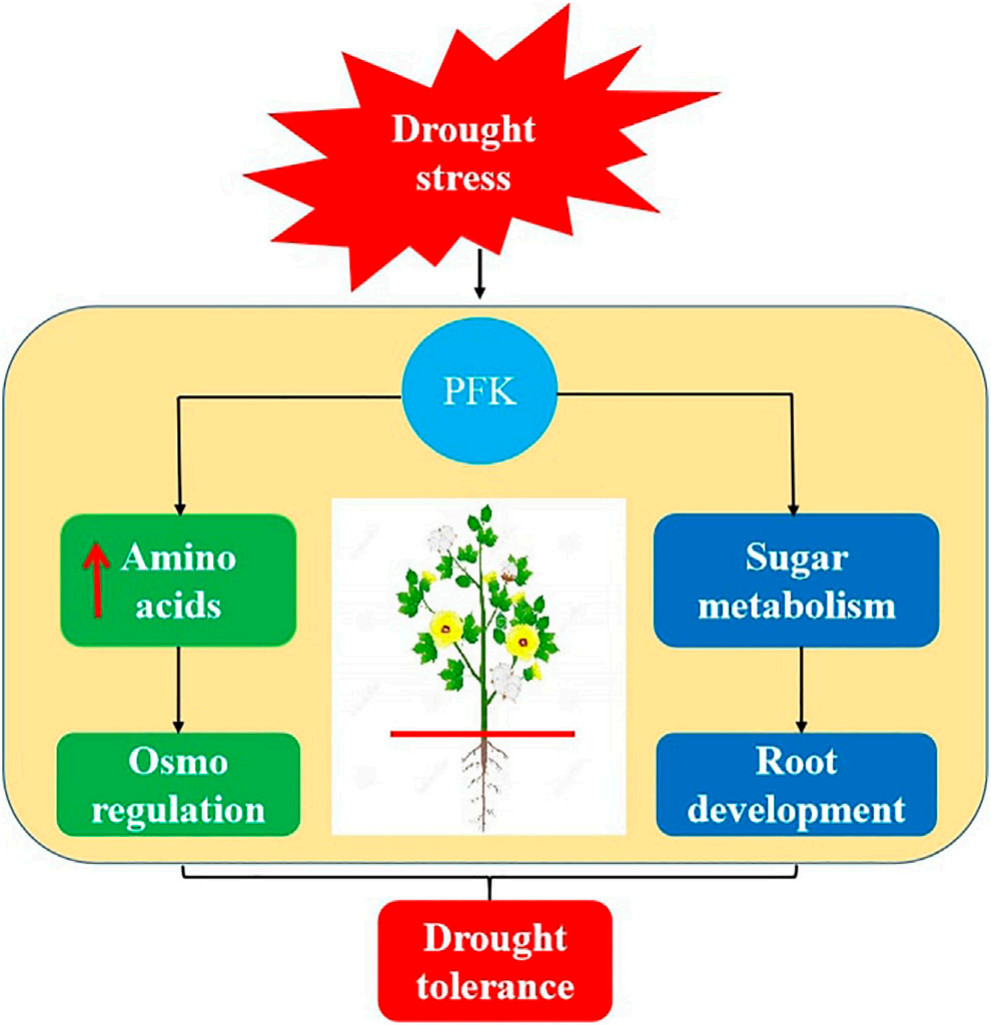
A graphic model for the role of PFK genes under drought stress. Drought stress induces biosynthesis and upregulation of amino acids production and activates the plant osmo regulation activity. Additionally, PFK involves in sugar metabolism and acts as the energy source for root development during plant drought stress tolerance.

The glycolysis and pentose phosphate pathways have a significant impact on crop tolerance under abiotic stress. Genome-wide identification, co-expression, RT-qPCR profiling, and metabolite enrichment analysis indicated that PFK gene family has potential in drought stress tolerance by involving in biosynthesis of amino acids and sugar metabolism activity (Figure 10). Important amino acids like L-valine, L-glutamine, L-methionine, and gluconic acid were significantly upregulated, while sugars like D-mannitol, D-sorbitol, and dulcitol were significantly downregulated during drought stress. The co-expression and RT-qPCR results showed that *GhPFK04*, *GhPFK09*, *GhPFK13*, *GhPFK14*, *GhPFK16*, and *GhPFK17* are hub genes and consistently upregulated in both leaf and root tissues during the stress condition. *GhPFK11* and *GhPFK17* were identified as common hub genes and these might be the true candidate genes involved in the drought stress tolerance in *G. hirsutum*. The current study lays the groundwork for the importance of PFK gene family in drought stress tolerance and sugar metabolism in cotton and opens doors to functional characterization of hub genes. Therefore, further functional validation of the hub genes is needed in order to better understand their involvement in the drought stress tolerance and sugar metabolism mechanisms at molecular and genetic levels.

## Data Availability

The datasets presented in this study can be found in online repositories. The names of the repository/repositories and accession number(s) can be found in the article/Supplementary Material.
