# Corrigendum: Genome-wide identification and expression analysis elucidates the potential role of PFK gene family in drought stress tolerance and sugar metabolism in cotton

**DOI:** 10.3389/fgene.2022.1052613

**Published:** 2022-10-10

**Authors:** Teame Gereziher Mehari, Yanchao Xu, Muhammad Jawad Umer, Fang Hui, Xiaoyan Cai, Zhongli Zhou, Yuqing Hou, Kai Wang, Baohua Wang, Fang Liu

**Affiliations:** ^1^ School of Life Sciences, Nantong University, Nantong, China; ^2^ State Key Laboratory of Cotton Biology, Cotton Institute of the Chinese Academy of Agricultural Sciences, Anyang, China; ^3^ School of Agricultural Sciences, Zhengzhou University, Zhengzhou, China

**Keywords:** cotton, phosphofructokinase, drought stress, sugar metabolism, RNA-Seq, RT-qPCR

In the published article, there was an error in the **Funding** statement. The funding information was not included in the final publication. The correct **Funding** statement appears below.

“This research was funded by the National Key R&D Program of China [(2021YFE0101200), PSF/CRP/18thProtocol (07)], the National Natural Science Foundation of China (32171994, 32072023).”

The authors apologize for this error and state that this does not change the scientific conclusions of the article in any way. The original article has been updated.

