# Supplementary material for: Genome-Wide Identification and Expression Analysis Elucidates the Potential Role of PFK Gene Family in Drought Stress Tolerance and Sugar Metabolism in Cotton
Source: Front Genet. 2022 Jun 20;13:922024. doi: 10.3389/fgene.2022.922024 (PMC9251378; doi:10.3389/fgene.2022.922024)
Supplement: Supplementary file 1 [file DataSheet1.ZIP › Supplementary table captions.docx]

**SUPPLEMENTARY TABLE CAPTIONS**

**Additional file 1: Supplementary Table S1.** List of primers for the RT-qPCR expression for *G. hirsutum* genes

**Additional file 2: Supplementary Table S2.** Sub-cellular localization prediction of the PFK gene family in *G. hirsutum*, *G. arboreum, G. raimondii* and *G. barbadense* species.

**Additional file 3: Supplementary Table S3.** List of cis regulatory elements, sequence and their description in *G. hirsutum*, *G. arboreum, G. raimondii* and *G. barbadense*.

**Additional file 4: Supplementary Table S4.** List of genes and their expression to drought stress at different time points and tissues in Marie galante-85, Upland cotton and Lattifolium-40 *Gossypium* races.

**Additional file 5: Supplementary Table S5.** Metabolic pathways, key metabolites and their relative expression in root and leaf tissues of in Marie galante-85, Upland cotton and Lattifolium-40 *Gossypium* races during drought stress
